# Circulating Angiopoietin-2 as a Biomarker in ANCA-Associated Vasculitis

**DOI:** 10.1371/journal.pone.0030197

**Published:** 2012-01-18

**Authors:** Paul A. Monach, Philipp Kümpers, Alexander Lukasz, Gunnar Tomasson, Ulrich Specks, John H. Stone, David Cuthbertson, Jeffrey Krischer, Simon Carette, Linna Ding, Gary S. Hoffman, David Iklé, Cees G. M. Kallenberg, Nader A. Khalidi, Carol A. Langford, Philip Seo, E. William St. Clair, Robert Spiera, Nadia Tchao, Steven R. Ytterberg, Marion Haubitz, Peter A. Merkel

**Affiliations:** 1 Section of Rheumatology, Department of Medicine, School of Medicine, Vasculitis Center, Boston University, Boston, Massachusetts, United States of America; 2 Department of Nephrology and Hypertension, Hannover Medical School, Hannover, Germany; 3 Division of General Internal Medicine, Nephrology, and Rheumatology, Department of Medicine, University Hospital Münster, Münster, Germany; 4 Division of Pulmonary and Critical Care Medicine, Mayo Clinic College of Medicine, Rochester, Minnesota, United States of America; 5 Rheumatology Unit, Massachusetts General Hospital, Boston, Massachusetts, United States of America; 6 Department of Pediatrics, College of Medicine, University of South Florida, Tampa, Florida, United States of America; 7 Division of Rheumatology, University of Toronto, Toronto, Canada; 8 National Institute of Allergy and Infectious Diseases, Bethesda, Maryland, United States of America; 9 Center for Vasculitis Care and Research, Cleveland Clinic, Cleveland, Ohio, United States of America; 10 Federal Systems Division, Rho, Chapel Hill, North Carolina, United States of America; 11 Department of Rheumatology and Clinical Immunology, University Medical Center, Groningen, The Netherlands; 12 Division of Rheumatology, McMaster University, Hamilton, Canada; 13 Division of Rheumatology, Johns Hopkins University, Baltimore, Maryland, United States of America; 14 Division of Rheumatology and Immunology, Duke University, Durham, North Carolina, United States of America; 15 Rheumatology Division, Hospital for Special Surgery, New York, New York, United States of America; 16 Immune Tolerance Network, San Francisco, California, United States of America; 17 Division of Rheumatology, Mayo Clinic College of Medicine, Rochester, Minnesota, United States of America; Beth Israel Deaconess Medical Center, United States of America

## Abstract

The endothelial-specific Angiopoietin-Tie2 ligand-receptor system is an important regulator of endothelial activation. Binding of angiopoietin-2 (Ang-2) to Tie2 receptor renders the endothelial barrier responsive to pro-inflammatory cytokines. We previously showed that circulating Ang-2 correlated with disease severity in a small cohort of critically ill patients with anti-neutrophil cytoplasmic antibody (ANCA)-associated glomerulonephritis. The current study reassessed Ang-2 as a biomarker of disease activity and relapse in AAV. Circulating Ang-2 was measured in 162 patients with severe AAV (BVAS/WG≥3, with or without glomerulonephritis) in a clinical trial. Ang-2 levels during active AAV were compared to levels in the same patients during remission (BVAS/WG = 0). Levels in clinical subsets of AAV were compared, and association with future disease course was assessed. Ang-2 levels were elevated in severe disease (median 3.0 ng/ml, interquartile range 1.9–4.4) compared to healthy controls (1.2, 0.9–1.5). However, they did not reliably decline with successful treatment (median 2.6 ng/ml, interquartile range 1.9–3.8, median change −0.1). Ang-2 correlated weakly with BVAS/WG score (r = 0.17), moderately with markers of systemic inflammation (r = 0.25–0.41), and inversely with renal function (r = −0.36). Levels were higher in patients with glomerulonephritis, but levels adjusted for renal dysfunction were no different in patients with or without glomerulonephritis. Levels were higher in patients with newly diagnosed AAV and lower in patients in whom treatment had recently been started. Ang-2 levels during active disease did not predict response to treatment, and Ang-2 levels in remission did not predict time to flare. Thus, Ang-2 appears to have limited practical value in AAV as a biomarker of disease activity at time of measurement or for predicting future activity.

## Introduction

ANCA-associated vasculitis (AAV) encompasses granulomatosis with polyangiitis (GPA, Wegener's) and microscopic polyangiitis (MPA), two diseases that used to have high fatality rates but are now successfully treated with immune-suppressive drugs. After induction of remission, disease course is highly variable, and available biomarkers such as titers of anti-neutrophil cytoplasmic antibodies (ANCA) and markers of systemic inflammation (ESR, CRP) do not provide adequate information about whether a patient is currently in remission or is at risk for relapse [1], [2], [3], [4], [5], [6], [7], [8]. In light of the pathophysiology of AAV, circulating proteins derived from damaged or activated microvascular endothelial cells are plausible candidates as biomarkers.

Angiopoietins are angiogenic factors essential for vascular development, maturation, and inflammation [9], [10], [11], [12]. As circulating or matrix-bound molecules, angiopoietin-1 (Ang-1) and angiopoietin-2 (Ang-2) bind to the extracellular domain of the tyrosine kinase receptor Tie2, predominantly expressed on endothelial cells [13], [14]. Constitutive Ang-1 expression by vascular mural cells, and low-level Tie2 phosphorylation, probably represent a non-redundant control pathway that maintains vessel integrity, prevents endothelial hyperpermeability and inhibits leukocyte-endothelium interactions [9], [15]. Upon a variety of stimuli, Ang-2 is rapidly released by the activated endothelium from Weibel-Palade bodies [16], disrupts constitutive Ang-1/Tie2 signalling by preventing Ang-1 from binding to Tie2 [13], [16], [17], and thereby promotes vascular permeability and leukocyte adhesion.

Circulating levels of Ang-2 are elevated in multiple disease states of endothelial activation and/or damage, such as sepsis [18], [19], [20], [21], systemic lupus erythematosus [22], and hypertension [23]. Elevated Ang-2 levels are associated with multi-system organ failure in acute pancreatitis [24] and with mortality in critically ill patients [19]. However, Ang-2 levels also rise moderately during progression of chronic kidney disease due to either IgA nephropathy or adult polycystic kidney disease [25], two diseases in which endothelial damage does not play a primary role.

In our previous study in AAV, serum levels of Ang-2 were much higher in 15 patients with untreated, severe AAV with glomerulonephritis (GN; 9 with GPA, 6 with MPA) than in three other groups: i) 20 patients with a history of AAV and GN but in remission and on minimal immune-suppressive medication at times of measurement; ii) 10 patients with active GPA limited to granulomatous disease of the respiratory tract but no evidence of systemic necrotizing vasculitis, off immune-suppressive drugs, and iii) 20 healthy age-matched controls [26]. In the 15 patients with active GN, Ang-2 levels did not correlate with glomerular filtration rate (GFR), indicating that kidney dysfunction per se was not the reason for Ang-2 elevation. Eight of these 15 patients also had samples available 6 months later, after successful treatment, and Ang-2 levels had returned to the normal range in 7 of these 8 patients.

Several questions were left unanswered by that pilot study. Are Ang-2 levels elevated in patients with severe AAV without GN? With treatment, how reliably do levels fall into the normal range? Do levels prior to treatment predict response to therapy? Do levels after achievement of remission predict future relapse? We addressed these questions using samples obtained from patients enrolled in the Rituximab in ANCA-Associated Vasculitis (RAVE) clinical trial [27], at times of severe active disease and remission, and in the Vasculitis Clinical Research Consortium (VCRC) Longitudinal Study, at times of mild disease, severe disease, or remission.

## Methods

### Study Subjects (RAVE, VCRC, and controls)

Subjects with AAV were enrolled in either the Rituximab in ANCA-Associated Vasculitis (RAVE) trial or the Vasculitis Clinical Research Consortium (VCRC) Longitudinal Study. Healthy control subjects were recruited at Boston University School of Medicine and at Medical College Hannover. All subjects were enrolled using protocols and written informed consent documents approved by local institutional review boards/ethics committees at all institutions at which participants were recruited and/or human subjects research was performed: Boston University, Cleveland Clinic, Duke University, Hannover Medical School, Hospital for Special Surgery, Johns Hopkins University, Mayo Clinic, McMaster University, University Medical Center Groningen, University of South Florida, and University of Toronto.

RAVE was a randomized, double-blinded, multi-center clinical trial of 197 patients with severe GPA or MPA that compared oral cyclophosphamide (CYC) to the B-cell-depleting agent rituximab (RTX) for induction of remission, each treatment combined with a standardized regimen of glucocorticoids [27]. All patients in the trial tested positive for antibodies to either proteinase 3 (PR3) or myeloperoxidase (MPO) at screening. Subjects randomized to receive CYC were switched to maintenance therapy with azathioprine (AZA) if they were clinically in remission between months 4 and 6. Subjects in the RTX arm were not placed on a maintenance agent but were considered to still be “on treatment” at month 6, since peripheral B cells were still undetectable in most patients. Prednisone was completely withdrawn before six months per study protocol. Investigators had the option to restart prednisone at no more than 10 mg/day to control recurrent symptoms of mild disease. Achievement of remission, with or without low-dose prednisone, was equivalent in the two treatment arms [27].

The VCRC Longitudinal Study is a multi-center, observational study of patients with six different forms of vasculitis, including GPA and MPA. Subjects were seen either quarterly or annually, and also at the time of flare of vasculitis, if possible. Blood samples were drawn at each visit and were linked to clinical data recorded on standardized forms.

### Patient Subgroups and Covariates (RAVE only)

The following subgroups were defined at the RAVE screening visit: i) GPA vs. MPA; ii) PR3-ANCA vs. MPO-ANCA; iii) active vs. inactive renal disease; and iv) newly diagnosed vs. relapsing disease. Initiation of treatment with glucocorticoids or other immune-suppressive drugs for severe AAV was allowed within 14 days preceding the screening visit for RAVE, and the presence or absence of such treatment was recorded using two dichotomous variables: any immune-suppressive drug, or specifically glucocorticoids. Covariates included age, sex, a diagnosis of hypertension, and a diagnosis of coronary artery disease.

### Selection of Patients and Processing of Samples (RAVE, VCRC, and controls)

For the RAVE cohort, each patient who had remained in the trial more than 4 months and from whom an adequate sample volume had been obtained at screening was included (n = 162). Among those 162 subjects, an additional sample from the month 6 visit was used only if the subject completed month 6 in the original treatment arm (n = 142). Subjects from the VCRC repository were chosen on the basis of having at least one visit at a time of active AAV (n = 68); additional samples were chosen from the same patients at times of either active AAV or remission if available. In total, 135 plasma samples from the 68 subjects were assayed, 1–6 samples per subject. Eighteen controls were available for assay of Ang-2 in serum, and 30 for assay of Ang-2 in plasma.

Serum (RAVE) and plasma (VCRC) were initially collected, processed, and stored at each study site, then shipped to central repositories, and then shipped to the study laboratory. All samples from RAVE remained frozen at −80°C until the day the assays were performed. Samples from the VCRC had been thawed and re-frozen once previously.

### Measurement of Vasculitis Disease Activity (RAVE and VCRC)

In RAVE, activity of AAV was measured using the Birmingham Vasculitis Activity Score for Wegener's Granulomatosis BVAS/WG [28], which summarizes disease activity in the past 28 days, and in which each severe disease manifestation is given 3 points and each non-severe manifestation is given 1 point. Remission was defined as BVAS/WG = 0, and active disease as BVAS/WG>0. In the VCRC, disease activity was assessed using the BVAS/WG and also by Physician Global Assessment (PGA) on a 0–10 Likert scale. In both studies, the presence or absence of items on the BVAS/WG that clearly indicate necrotizing vasculitis was noted.

### Longitudinal Outcome Measures (RAVE only)

Success of initial treatment was assessed by classifying subjects in three related ways. First, patients who remained in their original treatment groups, were in remission at month 6, and were off prednisone were compared to patients who did not meet these criteria. Second, patients in remission at month 6, including patients who were taking prednisone ≤10 mg/day, were compared to subjects who did not meet these criteria. Third, patients described as having a flare of AAV before month 6 were compared to those who did not have a flare recorded.

Time to flare after successful treatment (in remission at month 6) was assessed in two ways: time to severe flare (BVAS/WG≥3) and time to flare of any severity (BVAS/WG>0).

### Biomarker Assays and Additional Laboratory Data (RAVE and VCRC)

Circulating Ang-2 levels were measured by immuno-luminometric assay (ILMA, in RAVE samples) or ELISA (VCRC samples) using a pair of anti-Ang-2 monoclonal antibodies and recombinant Ang-2 as a standard (R&D, Oxon, U.K.) as described previously [26], [29]. VCRC samples and healthy control samples were assayed in duplicate, with the average of the two values used for analysis. The assays have a detection limit of 0.2 ng/mL. Inter-assay and intra-assay imprecision are <6%. Ang-2 is stable in serum and citrated plasma at room temperature for at least 24 h and is resistant to at least 4 freeze-thaw cycles. Levels in plasma are not significantly lower than in serum [29].

Westergren erythrocyte sedimentation rate (ESR), C reactive protein (CRP), and serum creatinine were assayed at the participating sites. In RAVE, GFR (in ml/min per 1.73 m^2^ body surface area) was estimated from serum creatinine (Cr, in mg/dL) using the MDRD formula [30].

Ang-2 levels in all samples from RAVE that were associated with estimated GFR<60 ml/min per 1.73 m^2^ body surface area were also adjusted for the effect of low GFR based on results of linear regression (see below).

### Statistical Analysis

#### Distributions of Marker Values, and Adjustment for Multiple Comparisons

Distributions of Ang-2 values were evaluated for normality using Shapiro-Wilk and Kolmogorov-Smirnov tests, as well as visual inspection of histograms. Since Ang-2 levels were not normally distributed among subjects with active vasculitis in RAVE, all data are reported as medians and interquartile ranges and were analyzed using non-parametric statistics. Findings in all analyses were considered significant at P<0.05, after adjustment for multiple comparisons [31]. All analyses were performed using SAS 9.1 (SAS Institute, Cary, NC).

#### Comparing Active AAV to Remission or to Healthy Controls (RAVE and VCRC)

In RAVE, change in Ang-2 level from screening to month 6 among patients in remission at month 6 was analyzed by Wilcoxon Signed Rank test. Ang-2 levels adjusted for the effect of low GFR were also analyzed in this way. Correlation of (unadjusted) Ang-2 level with BVAS/WG score at screening was analyzed using Spearman coefficients. Comparisons of Ang-2 levels between i) patients at screening and healthy controls, ii) patients in remission at month 6 and healthy controls, and iii) patients with recurrent or persistently active disease at month 6 and patients in remission, were analyzed by Wilcoxon Rank Sum tests.

In the VCRC, Ang-2 levels in samples associated with active AAV, AAV in remission, and healthy controls were compared by Wilcoxon Rank Sum tests. Correlation of Ang-2 level with BVAS/WG score or PGA was measured by Spearman coefficients. Since patients contributed 1–6 samples and had variable numbers of visits characterized by active disease or remission, association of Ang-2 with active AAV was also tested using generalized linear models (GLM) in which patient identification numbers were included along with disease activity measures (active disease or remission, or BVAS/WG score, or PGA) as independent variables modeling Ang-2 level as the dependent variable.

#### Clinical Subsets, Covariates, and Laboratory Parameters (RAVE only)

Marker levels at screening in dichotomous subgroups were compared using Wilcoxon Rank Sum tests (GPA vs. MPA, PR3-ANCA vs. MPO-ANCA, male vs. female, new vs. relapsing disease, treated vs. untreated, and presence vs. absence of active renal disease, alveolar hemorrhage, hypertension, or coronary artery disease). Ang-2 levels in some of these subgroups (GPA/MPA, ANCA specificity, new/relapsing, treated/untreated, active renal disease, or alveolar hemorrhage) were also analyzed after adjustment for effects of low GFR.

Correlation of Ang-2 levels with continuous variables (ESR, CRP, age or GFR) was assessed using Spearman coefficients, in all subjects at screening or in subjects in remission at month 6. Linear regression was used to estimate the average change in Ang-2 associated with rise in age and/or decline in GFR (age and/or GFR as independent variable(s), Ang-2 level as dependent variable). The beta coefficient of the linear regression of Ang-2 vs. GFR among subjects in remission at month 6 in RAVE was used to adjust Ang-2 levels for GFR<60 ml/min per 1.73 m^2^, as noted above. Spearman correlation of Ang-2 levels with ESR and CRP was also measured in the VCRC cohort.

#### Post-hoc Analysis of Subjects with Untreated, Newly-Diagnosed AAV with Renal Involvement, and of Subjects with the Highest Ang-2 Levels (RAVE only)

Subjects in RAVE who most closely resembled the subjects in our previous study [26] were selected in three ways. First, Ang-2 levels in subjects with newly-diagnosed AAV who had not yet been treated were compared to subjects not meeting these criteria, by Wilcoxon Rank Sum test. Second, Ang-2 levels in subjects with newly-diagnosed AAV including GN who had not yet been treated were compared to subjects not meeting these criteria. Third, the clinical features of the 15 subjects with Ang-2>6 ng/ml at screening were compared to those of the 147 subjects with lower levels. Age, GFR, and BVAS/WG score were compared by Wilcoxon Rank Sum tests, and the frequencies of GPA (vs. MPA), newly-diagnosed AAV (vs. relapsing), current treatment (vs. untreated), active GN, and active alveolar hemorrhage were compared by Fisher Exact tests.

#### Prediction of Response to Treatment, or of Future Relapse (RAVE only)

For prediction of response to treatment, Ang-2 levels at screening were compared, using Wilcoxon Rank Sum tests, in groups defined by three related outcomes at month 6, as defined above.

For prediction of future relapse in RAVE, analysis was limited to subjects in remission, in their original treatment groups, and off prednisone at month 6 (n = 104). Ang-2 level at month 6, treatment group (CYC/AZA or RTX), ANCA specificity, new or relapsing disease at screening, age, and sex were used as independent variables to predict two separate outcomes (time to severe flare, and time to any flare) as defined above. All variables associated individually with time to flare at P<0.2 were then included as covariates alongside Ang-2 in multivariable Cox proportional hazards models.

## Results

### Ang-2 in Active AAV and Remission (RAVE and VCRC)

All 162 subjects from RAVE who were included in the current study had severe disease at screening, with median BVAS/WG = 8, interquartile range 6–10 (**Table 1**). One hundred thirty-eight patients had at least one manifestation on the BVAS/WG that indicated necrotizing vasculitis rather than merely a combination of granulomatous, musculoskeletal, and constitutional features. Serum Ang-2 levels in these 162 patients were higher than in 18 healthy controls: median 3.0 (interquartile range 1.8–4.4) vs. 1.2 (0.9–1.5) ng/ml, P<0.0001.

**Table 1:** Demographic and disease-related features of the patients in this study.

	RAVE (n = 162)	VCRC (n = 68)
Age*	52 (44;66)	52 (36;60)
Sex (female)	83 (51%)	40 (59%)
AAV subtype (GPA)	126 (78%)	65 (96%)
**Active disease samples**	162†	81
Active treatment	89 (55%)	69 (85%)
Glucocorticoid treatment	78 (48%)	64 (79%)
BVAS/WG*	8 (6;10)	2 (1;4)
Necrotizing vasculitis	138 (85%)	26 (32%)
**Remission samples**	120	54
Active treatment	120 (100%)	44 (81%)
Glucocorticoid treatment	15 (13%)	38 (70%)

GPA = granulomatosis with polyangiitis.

*Median (interquartile range).

† Data shown regarding active disease are limited to samples at screening. Twenty-two of these patients also had active disease of lower severity at month 6: median BVAS/WG = 1 (interquartile range 1–2).

**Figure 1:**
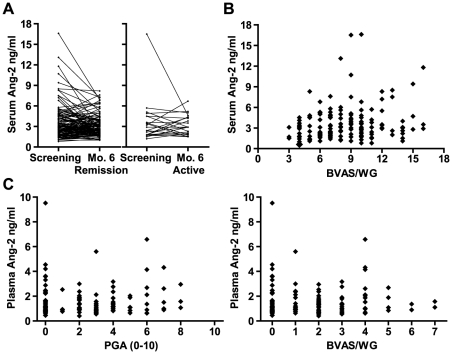
Angiopoietin-2 (Ang-2) levels at different levels of disease activity in ANCA-associated vasculitis. **A.** Serum levels in individual patients in the RAVE trial before (Screening) and after (Month 6) treatment, stratified by whether patients were in remission (left panel) or had recurrent disease (right panel) at month 6. **B.** Plot of serum Ang-2 versus BVAS/WG score in individual subjects in RAVE at screening. Each patient had severe disease and therefore a BVAS/WG score of at least 3. **C.** Plot of plasma Ang-2 versus two measures of disease activity in the VCRC longitudinal study: Physician Global Assessment (PGA, left panel) and BVAS/WG score (right panel). Note that patients in remission (PGA = 0 and BVAS/WG = 0) are included, and that Ang-2 levels are lower in plasma than in serum.

One hundred forty-two of these 162 subjects completed 6 months in their original treatment groups and had samples available at month 6; of those 142, 120 were in remission and 22 had recurrent or persistent, usually mild disease (median BVAS/WG = 1, interquartile range 1–2). Among the 120 subjects in remission at month 6, there was no significant difference in serum Ang-2 between screening and remission. The median decrease was 0.1 ng/ml with a broad range (**Table 2** and **Figure 1A**), and levels remained higher than among healthy controls (P<0.0001). Results were similar among the 22 subjects who had recurrent or persistent disease at month 6 (**Table 2** and **Figure 1A**).

**Table 2:** Ang-2 levels in patients with severe active ANCA-associated vasculitis and in the same patients after treatment in the RAVE trial.

Clinical Status at Month 6	Screening	Month 6	Difference, Screening minus Month 6	P*
Remission (n = 120)	3.0 (1.9;4.4)	2.6 (1.9;3.8)	0.1 (−0.7;1.3)	0.099
Active (n = 22)	2.7 (1.8;4.5)	2.9 (1.9;4.3)	0.05 (−0.8;0.7)	0.99

All values indicate serum Ang-2 in ng/ml; medians and interquartile ranges are shown.

*Comparing the difference (screening minus month 6) to the null distribution (Wilcoxon Signed Rank test); P = 0.57 comparing the differences seen in Month 6 Remission and Month 6 Active, P = 0.6 comparing Remission with Active at month 6 (Wilcoxon Rank Sum test).

The VCRC cohort included 81 samples from 68 subjects at times of active disease: median BVAS/WG = 2, interquartile range 1–4, full range 1–7 (**Table 1**). Among these 81 samples, 26 were associated with necrotizing vasculitis, and the other 55 with granulomatous, musculoskeletal, and/or constitutional manifestations. Thus, disease severity was lower than for the patients in RAVE at screening visits. Fifty-four samples from among the same 68 patients at times of remission were also measured. Plasma Ang-2 levels were no higher in samples taken at times of active disease (median 1.2 [interquartile range 0.9–1.9] ng/ml) than in samples taken during remission (1.1 [0.8–1.7] ng/ml; P = 0.33) or from 30 healthy controls (1.1 [0.8–1.4] ng/ml; P = 0.08). However, Ang-2 levels were somewhat higher in the 26 samples associated with necrotizing vasculitis (1.6 [1.1;2.6] ng/ml) than in the 55 samples representing only non-vasculitic manifestations (1.1 [0.8;1.9] ng/ml; P = 0.03) or in the 54 samples reflecting remission (1.1 [0.9;1.4] ng/ml; P = 0.004).

Among the 162 patients at screening in RAVE, there was a statistically significant but weak association between serum Ang-2 and BVAS/WG: r = 0.17, P = 0.03 (**Figure 1B**). Because the BVAS/WG reflects disease activity in the past 28 days, and some patients in RAVE had begun treatment within the past 14 days, a separate analysis was performed limited to untreated patients, in whom the BVAS/WG score clearly indicates disease activity at the time of sample collection. The association of Ang-2 with BVAS/WG in these 73 patients was unchanged: r = 0.18. In the VCRC, there was no significant correlation between plasma Ang-2 and either BVAS/WG score (r = 0.11, P = 0.20) or PGA (r = 0.13, P = 0.15; see **Figure 1C**). Restricting the correlation analysis to subjects with active disease (BVAS/WG>0 and PGA>0) did not change the results, nor did accounting for the presence of variable numbers of active and remission visits from different subjects (data not shown, see Methods).

### Ang-2 and Markers of Systemic Inflammation (RAVE and VCRC)

**Figure 2:**
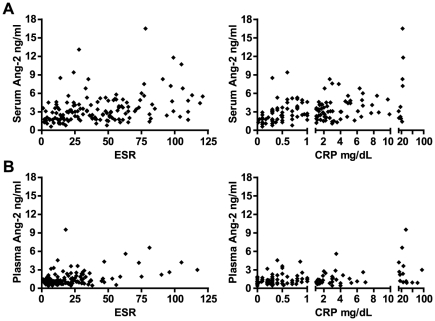
Plots of angiopoietin-2 (Ang-2) levels versus markers of systemic inflammation. **A.** Serum Ang-2 versus ESR (left panel) and CRP (right panel) in the RAVE trial. Values at screening, when each patient had severe disease (BVAS/WG≥3), are shown. **B.** Plasma Ang-2 versus ESR (left panel) and CRP (right panel) in the VCRC longitudinal study. Note that patients in remission (BVAS/WG = 0) are included, and that Ang-2 levels are lower in plasma than in serum.

As shown in **Figure 2A**, among the 162 subjects with active AAV at screening in RAVE, serum Ang-2 correlated moderately well with ESR (r = 0.38, P<0.0001) and CRP (r = 0.35, P<0.0001). In the VCRC, correlation of plasma Ang-2 with ESR and CRP were of similar magnitude (**Figure 2B**), regardless of whether analysis was restricted to the 81 samples associated with active disease (r = 0.41 and 0.33, P = 0.0005 and P = 0.008, respectively) or included the 54 remission samples (r = 0.35 and 0.25, P<0.0001 and P = 0.008, respectively).

### Ang-2 in Subsets of AAV (RAVE only)

The 162 subjects in RAVE at screening were classified according to AAV subtype (GPA or MPA), ANCA specificity, presence or absence of renal disease or alveolar hemorrhage, and newly diagnosed or established AAV. Ang-2 levels were higher in patients with active renal disease or with a new diagnosis of AAV, although differences were modest (**Table 3**).

**Table 3:** Ang-2 levels in clinical subgroups of active ANCA-associated vasculitis in the RAVE trial.

Subgroups*	N†	Yes‡	No‡	P
GPA (vs. MPA)	126,36	2.9 (1.8;4.2)	3.1 (2.15;5.4)	0.20
PR3 ANCA (vs. MPO)	112,50	2.95 (1.8;4.2)	3.0 (2.1;4.6)	0.56
Renal Disease	79,83	3.1 (2.2;4.4)	2.5 (1.7;4.5)	0.042
Alveolar Hemorrhage	42,120	2.7 (1.9;3.5)	3.05 (1.8;4.5)	0.25
New Diagnosis	77,85	3.5 (2.4;5.3)	2.4 (1.7;3.4)	0.0001

*All data (clinical subgroups and Ang-2 levels) were obtained at screening. GPA = granulomatosis with polyangiitis; MPA = microscopic polyangiitis; PR3 = proteinase 3; MPO = myeloperoxidase.

† Numbers of subjects fulfilling and not fulfilling the subgroup designation.

‡ Serum Ang-2 (ng/ml) in subjects fulfilling (Yes) and not fulfilling (No) the subgroup designation; medians and interquartile ranges are shown.

**Figure 3:**
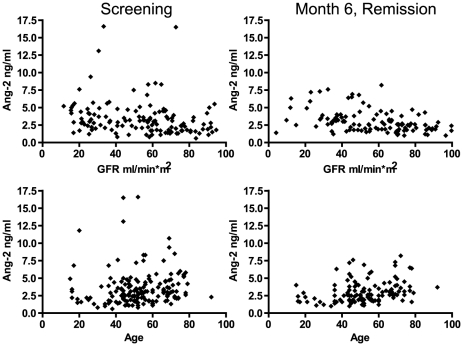
Plots of serum angiopoietin-2 (Ang-2) levels versus GFR (top panels) or age (bottom panels). Samples from subjects in RAVE were assayed at times of active AAV (screening, left panels) and remission 6 months after starting treatment (right panels).

In order to address the possibility that impaired renal function had a direct effect on Ang-2 levels, association of Ang-2 with GFR was measured not only among subjects at screening (median 60 ml/min/1.73 m^2^, interquartile range 36–79), but more importantly among subjects in remission at month 6 (median 61, interquartile range 41–74), so as to avoid active GN as a confounder. A significant negative association was found particularly among subjects in remission (r = −0.23, P = 0.005 at screening; r = −0.36, P<0.0001 at month 6; **Figure 3**), corresponding to an average increase in serum Ang-2 of 0.18 ng/ml (± SE 0.05) per 10 ml/min/1.73 m^2^ reduction in GFR using the data at month 6. Re-analysis of changes in Ang-2 levels after treatment and of Ang-2 levels in clinical subgroups at screening using Ang-2 levels adjusted for the effect of low GFR changed only one of the conclusions to be drawn from Tables 1, 2, and 3: subjects with active GN no longer had higher values than subjects with active AAV without GN (median 2.8 vs. 2.5 ng/ml, P = 0.33).

### Effects of Treatment, GFR, and Other Covariates (RAVE only)

Subjects who were already being treated with glucocorticoids and/or other immune-suppressive drugs at the time of screening in RAVE had lower levels of Ang-2 than did patients who had not yet been treated (**Table 4**). Doses of glucocorticoids were not available but were presumed to be at or near 1 mg/kg since these patients were being treated for severe flares, and duration of such treatment was no more than 2 weeks per study protocol. Among patients in remission at month 6, levels did not differ between patients treated with CYC/AZA versus those treated with RTX, but were lower among the small number of patients in whom glucocorticoids had been restarted (**Table 4**).

**Table 4:** Effects of treatment or covariates on Ang-2 levels in the RAVE trial.

Treatment at Screening*	N§	Yes¶	No¶	P
On Treatment	89,73	2.7 (1.8;3.7)	3.1 (2.3;5.0)	0.011
On Glucocorticoids	78,84	2.8 (1.7;3.7)	3.1 (2.2;4.7)	0.033

Treatment at month 6†	N§	Yes¶	No¶	P
RTX (vs. CYC/AZA)	65,50	2.5 (1.8;3.7)	2.8 (2.1;3.9)	0.28
On Prednisone	15,105	1.9 (1.4;2.5)	2.7 (2.0;3.9)	0.007

Covariate‡	N§	Yes¶	No¶	P
Hypertension	41,79	3.0 (1.9;4.4)	2.6 (1.9;3.5)	0.22
Coronary Artery Disease	7,113	2.6 (1.8;3.9)	2.6 (1.9;3.8)	0.93
Male (vs. Female)	58,62	2.6 (1.9;3.9)	2.6 (1.9;3.8)	0.96

*Values at screening. “On Treatment” = glucocorticoids or any other immune-suppressive drug.

† Values at month 6 restricted to subjects in remission. RTX = rituximab. CYC/AZA = cyclophosphamide followed by azathioprine. Only subjects who were still in their original treatment groups were included in the RTX vs. CYC/AZA analysis.

‡ Values at month 6 restricted to subjects in remission.

§ Numbers fulfilling and not fulfilling the criteria in column one.

¶ Serum Ang-2 (ng/ml) in subjects fulfilling (Yes) and not fulfilling (No) the criteria in column one; medians and interquartile ranges are shown.

Other covariates were assessed among subjects in remission at month 6 so as to eliminate active AAV as a contributor to Ang-2 levels. Levels did not differ between subjects with or without diagnoses of coronary artery disease or hypertension, nor between men and women (**Table 4**). Ang-2 levels increased significantly with age (r = 0.23, P = 0.003 at screening; r = 0.34, P = 0.0002 at month 6; **Figure 3**), corresponding to an average increase of 0.29 ng/ml per 10 years of age using the data at month 6. Because age and GFR were negatively correlated with each other (r = −0.39, P<0.0001 at screening; r = −0.42, P<0.0001 at month 6), multiple linear regression was performed, which suggested that age and GFR provided some independent information but were predominantly measuring the same predictive factor: P = 0.04 for each variable and R^2^ = 0.11 in the combined model, compared to P = 0.002 and R^2^ = 0.08 for either variable individually.

### Post-hoc Analysis of Subsets Resembling the Previously-Studied Cohort (RAVE only)

Because these results differed from those in our previous study [26], an additional analysis of RAVE patients at screening was limited to those who more closely resembled those in the earlier report: patients with newly-diagnosed AAV and not yet treated. The 57 subjects who met these criteria had median Ang-2 of 3.2 ng/ml (interquartile range 2.4–5.5), compared to 2.8 (1.8–3.7) ng/ml for the 105 subjects not meeting these criteria, P = 0.002. Further restricting the subset to newly-diagnosed, untreated patients with active GN had little if any effect: the 33 subjects who met these criteria had median Ang-2 of 3.7 (2.2–4.7) ng/ml, compared to 2.9 (1.8–4.1) ng/ml for the 129 subjects not meeting these criteria, P = 0.07.

The subset of 15 patients with Ang-2>6 ng/ml at screening was also compared to the 147 patients with lower levels. This subset had higher BVAS/WG scores and was more likely to have newly-diagnosed and untreated disease, but was not significantly more likely to have GN (**Table S1**).

### Ang-2 and Prediction of Response to Treatment or Duration of Remission (RAVE only)

Serum Ang-2 levels at screening in RAVE were no different in patients who i) did or did not achieve remission off prednisone at month 6, ii) did or did not achieve remission by month 6 on no more than 10 mg/day prednisone, or iii) did or did not have a flare of AAV before month 6 (**Table 5**).

**Table 5:** Lack of association of Ang-2 levels during active AAV with response to treatment in the RAVE trial.

Outcome*	N†	Yes‡	No‡	P
Primary Endpoint	98,59	3.0 (1.9;4.4)	2.9 (1.8;4.4)	0.63
Remission	122,35	3.0 (1.9;4.4)	2.6 (1.8;3.8)	0.44
Flare	33,124	2.9 (1.8;4.5)	2.9 (1.9;4.2)	0.85

*Primary Endpoint = completed month 6 per study protocol, in remission and not on prednisone. Remission = in remission at month 6 regardless of whether primary endpoint was met. Flare = worsening disease, or active disease following a period of remission, during the first six months of treatment.

† Numbers fulfilling and not fulfilling the criteria in column one.

‡ Serum Ang-2 (ng/ml) in subjects fulfilling (Yes) and not fulfilling (No) the criteria in column one; medians and interquartile ranges are shown.

Although Ang-2 levels among patients in remission were higher than in healthy controls (see above), these levels were not predictive of time to severe flare (BVAS/WG≥3) or time to flare of any severity, either as the only predictive variable or in combination with other clinical variables associated with risk of relapse (**Table 6**). Results were similar regardless of whether Ang-2 level was treated as an untransformed or log-transformed variable (data not shown).

**Table 6:** Lack of association of Ang-2 level during remission (and off prednisone) with time to flare of ANCA-associated vasculitis (AAV) in the RAVE trial.

	HR (95% CI), time to any flare †		HR (95% CI), time to severe flare ‡	
Variable*	Univariable	Multivariable	Univariable	Multivariable
Age (years)	0.995 (0.977–1.01)	ND	0.985 (0.965–1.01)	0.987 (0.965–1.01)
RTX treatment	1.76 (0.970–3.19)	1.61 (0.882–2.95)	1.22 (0.630–2.38)	ND
PR3 ANCA	1.91 (0.998–3.67)	1.61 (0.828–3.12)	2.00 (0.914–4.38)	1.80 (0.814–3.98)
Relapsing AAV	1.97 (1.12–3.48)	1.71 (0.958–3.05)	1.81 (0.937–3.49)	1.62 (0.828–3.15)
Ang-2 (ng/ml)	0.923 (0.767–1.11)	0.963 (0.796–1.17)	0.909 (0.729–1.14)	0.987 (0.776–1.25)

*Variables were continuous for age and Ang-2, dichotomous for the others; RTX = rituximab (referent = cyclophosphamide/azathioprine); referent for PR3 ANCA is MPO ANCA; relapsing AAV refers to status at screening (referent = new diagnosis of AAV).

† HR = hazard ratio, CI = confidence interval, using Cox proportional hazards models; ND = not done because P value of covariate in univariable model was >0.2 (Ang-2 was included in all models).

‡ Severe flare = BVAS/WG≥3.

## Discussion

This large study of patients with AAV, followed longitudinally so that samples were obtained during both active disease and remission, confirmed that circulating Ang-2 levels are elevated during severe active AAV but not mild AAV, but failed to confirm that levels decline after 6 months of successful treatment [26]. Furthermore, levels during active disease were not predictive of success or failure of treatment, and levels during remission were not predictive of time to flare.

Sub-group analyses help to reconcile the current results with the more striking findings of our previous report, in which all patients had active GN, half were newly diagnosed and not yet treated, and the half who were having relapses were either untreated or only on low dose glucocorticoids [26]. Similarly, in the current study, subjects with GN had higher Ang-2 levels than those without GN; subjects with newly-diagnosed AAV had higher levels than subjects with relapsing AAV; subjects who were untreated had higher levels than subjects already on an immune-suppressive drug at the time of screening; and untreated subjects with newly-diagnosed AAV had higher levels than subjects not meeting these criteria. In addition, the extent of renal impairment among patients with GN was somewhat lower in the current study than in the previous one: mean GFR 62±36 ml/min compared to 43±28 ml/min in the previous study (using the Cockcroft-Gault formula to allow comparison).

Indeed, analyses of Ang-2 and GFR in patients clinically in remission suggested that low GFR had an effect on Ang-2 levels, as we have previously observed in patients with chronic kidney disease due to IgA nephropathy or polycystic kidney disease [25]. We have previously discussed why this association is more likely to be related to endothelial dysfunction rather than reduced clearance of Ang-2 [25]. Reduced GFR probably contributes to elevation of Ang-2 in active AAV and may be the only reason that patients with GN have higher levels than patients without GN. However, GFR is not the driving force for elevation of Ang-2 in severe AAV overall based on the data shown in Figure 3 and our previous finding that Ang-2 did not correlate with GFR in severe AAV with GN [26]. Similarly, the persistent elevation of Ang-2 in many patients clinically in remission cannot be explained by chronic kidney disease with reduced GFR, since adjustment of Ang-2 levels for low GFR did not change the median value (2.6 ng/ml) in such patients.

Other markers of endothelial activation or damage have been reported to be elevated in patients with active AAV, including endothelial microparticles [32], intercellular adhesion molecule-1 (ICAM-1) [33], [34], [35], [36], [37], thrombomodulin [38], [39], vascular cell adhesion molecule-1 (VCAM-1) [33], [34], [36], [37], [40], and von Willebrand factor [38]. These studies have also reported either decline in marker level after treatment, and/or significantly lower levels among different patients with AAV in remission, and/or correlation with indices of disease activity, as we also reported in our pilot study of Ang-2 [26]. However, in several studies, the ranges of values among patients in remission appeared to be broader than the ranges in healthy controls [32], [35], [36], [39], as we have found for Ang-2 in the previous [26] and current studies and also for thrombomodulin [41] and ICAM-1 (Monach et al, unpublished data) using data after 6 months of treatment in the RAVE trial. It remains unclear whether persistent elevation of such markers has any implications regarding risk of relapse or progression of vascular disease by other mechanisms.

This study suggests that treatment with immune-suppressive drugs may lead directly and rapidly to reduction in Ang-2 levels, based on comparison of treated and untreated subjects at screening in RAVE and on comparison of patients in remission at month 6 who were or were not taking prednisone. One limitation of this study is that this suggestion cannot be directly tested due to the absence of short-term longitudinal data on previously untreated patients. Conversely, the strengths of this study include the use of two relatively large, prospective cohorts with well-defined and diverse manifestations of AAV, and the use of a treatment protocol in the larger of those cohorts.

Our data indicate that circulating Ang-2 levels have little promise as a clinically useful biomarker in AAV. Although Ang-2 is elevated in other vasculopathic conditions [19], [22], [23], [29], Ang-2 levels have not been shown to differentiate among vascular diseases. The current study also suggests that Ang-2 levels are not useful in AAV to gauge prognosis or assess efficacy of intervention. Finally, although Ang-2 levels in AAV probably reflect to some extent the burden of disease (i.e., in a broad range along the continuum of values reported in hypertension, systemic lupus erythematosus, and sepsis, and as reflected in correlation with ESR, CRP, and BVAS/WG values), this effect is not sufficiently strong to distinguish between severe AAV and remission. The possibility that persistent elevation of Ang-2 after induction of remission reflects vascular damage that could predispose to progression of renal insufficiency or atherosclerosis remains a viable and testable hypothesis.

## Supporting Information

Table S1 * Medians and interquartile ranges, or proportions of subjects fulfilling the subgroup designation.
(DOC)
