# Supplementary material for: Circulating Angiopoietin-2 as a Biomarker in ANCA-Associated Vasculitis
Source: PLoS One. 2012 Jan 18;7(1):e30197. doi: 10.1371/journal.pone.0030197 (PMC3261176; doi:10.1371/journal.pone.0030197)
Supplement: Table S1 — * Medians and interquartile ranges, or proportions of subjects fulfilling the subgroup designation. (DOC) [file pone.0030197.s001.doc]

**Table S1. Characteristics of subjects in the RAVE trial stratified by serum Ang-2 levels at screening.**

| **Clinical or Demographic Variable** | **Ang-2 > 6 ng/ml*(n=15)** | **Ang-2 ≤ 6 ng/ml*(n=147)** | **P** |
| --- | --- | --- | --- |
| Age (years) | 52 (41,66) | 52 (44,66) | 0.63 |
| GFR (ml/min*m^2^) | 58 (33,73) | 61 (37,79) | 0.93 |
| BVAS/WG | 10 (8,13) | 8 (6,10) | 0.02 |
| GPA (vs. MPA) | 10/15 | 116/147 | 0.33 |
| Alveolar Hemorrhage | 1/15 | 41/147 | 0.12 |
| Renal Disease | 6/15 | 73/147 | 0.59 |
| New Diagnosis | 13/15 | 64/147 | 0.002 |
| On Treatment | 4/15 | 85/147 | 0.03 |
| New Diagnosis, Untreated | 10/15 | 47/147 | 0.01 |
| New, Untreated, Renal Disease | 4/15 | 29/147 | 0.51 |

* Medians and interquartile ranges, or proportions of subjects fulfilling the subgroup designation.
